# Assessing the Knowledge Level, Attitudes, Risky Behaviors and Preventive Practices on Sexually Transmitted Diseases among University Students as Future Healthcare Providers in the Central Zone of Malaysia: A Cross-Sectional Study

**DOI:** 10.3390/ijerph14020159

**Published:** 2017-02-08

**Authors:** Adigun Temiloluwa Folasayo, Afolayan John Oluwasegun, Suhailah Samsudin, Siti Nor Sakinah Saudi, Malina Osman, Rukman Awang Hamat

**Affiliations:** 1 Department of Medical Microbiology and Parasitology, Faculty of Medicine and Health Sciences, Universiti Putra Malaysia, 43400 Serdang, Selangor, Malaysia; temmygraces@gmail.com (A.T.F.); suhailah.sam@gmail.com (S.S.); sakinahsaudi@gmail.com (S.N.S.S.); malinaosman@upm.edu.my (M.O.); 2 School of Architectural Design (SOAD), Linton Universiti College, Persiaran UTL, Bandar Universiti Teknologi Legenda (BUTL), Batu 12, 71700 Mantin, Negeri Sembilan, Malaysia; jamsxz2001@yahoo.com

**Keywords:** sexually-transmitted diseases, university students, future health providers, sexual behavior

## Abstract

This study was done to assess the knowledge, attitudes, risky behaviors and preventive practices related to sexually-transmitted diseases (STDs) among health and non-health sciences university students as future healthcare providers in Malaysia. A total of 700 health and non-health sciences university students (255 male; 445 female) aged between 17 and 30 years were surveyed by using a self-administered questionnaire. The majority (86.6%) had heard of STDs, and 50.4% knew STDs could present without symptoms. HIV remains the best known STD (83.6%) by the students, while chlamydia (26%) and trichomoniasis (21.0%) were rarely known. Gender, age group, educational level and faculty type were strongly associated with knowledge level (*p*-values < 0.05). Most of them (88.8%) were aware that STD screening was important while use of condoms was protective (63.8%). The majority of them strongly felt that treatment should be sought immediately if they (85.5%) and their partners (87.4%) have symptoms. Among the sexually-active students, 66.7% and 18% had sexual intercourse with multiple partners and commercial sex workers, while 17.4% and 9.4% took alcohol and drugs before having sex, respectively. By logistic regression analysis, students aged 24–30 years old (an odds ratio (AOR) = 0.57, 95% confidence interval (CI) = 0.377–0.859) and faculty type (AOR = 5.69, 95% CI = 4.019–8.057) were the significant predictors for the knowledge level. Knowledge on the non-HIV causes of STDs is still lacking, and the risky behavior practiced by the sexually-active students in this study is alarming. There is a need to revisit the existing STD education curriculum in both schools and universities so that appropriate intervention on STDs can be implemented.

## 1. Introduction

Sexually-transmitted diseases (STDs) have remained an important global health issue with an estimated one million people acquiring new infections every day, making up a total of about 499 million new cases of curable infections each year [1]. Globally, youths shoulder the burden of more than half of all new STDs annually [2,3].

In Malaysia, 70,559 cases of STDs were reported by UNAIDS, out of which 10,663 cases were AIDS, while more than 50,000 cases were for other STDs [4]. The magnitude of the STD problem is still unknown due to under-reporting, under-diagnosis or asymptomatic manifestation of these diseases. However, a previous report has proposed the rising propensity of STDs among the general population especially for early syphilis, herpes and genital warts, although the exact data for each type of STD was not available [5]. Moreover, the incidence of premarital sex has increased over the years. A report showed that 8.3% of school students have already had sex with the first sexual encounter at the mean age of 15 years [6]. Most of these students lacked awareness of the possible consequences of their actions, and the majority of them had had unprotected sex with multiple partners. A series of knowledge, attitudes and practices (KAP) studies related to STDs has been carried out in Malaysia; but the majority of them have focused mainly on HIV/AIDS, and schoolchildren have been the main target group [6,7,8,9].

Combating STDs among young adults is a daunting task for health professionals in most countries. Overcoming medical and social issues related to STDs will be more difficult if future healthcare providers are not well equipped with sound knowledge, good attitudes and practices. University students, medical and health sciences-based students in particular, are considered future healthcare providers who may be in a position to promote health education on STDs and to implement appropriate preventive measures for the public at large. They are thought to be more enlightened and well informed compared to the general population [10] and are expected to possess good KAP towards STDs. Thus, it is interesting to explore their level of knowledge, attitudes and practices on STDs so that appropriate interventions can be rectified or planned accordingly in the future.

## 2. Materials and Methods

### 2.1. Sample

The study was conducted in the central zone of Malaysia based on logistic purposes. Peninsular Malaysia is divided into four zones, namely the northern zone, central zone, southern zone and east coast. The central zone of Malaysia has only two states, which are the states of Selangor and Negeri Sembilan. Since the study involved future healthcare providers, two higher-learning institutions, which represent both public and private universities, were selected from this zone based on the medical and health sciences courses that were offered in those institutions. Selangor is bounded to the north and east by the state of Perak, and it is bordered by Negeri Sembilan to the south. The selected public university has 16 faculties, 9 institutes, 16 centers, 1 school and 1 academy with 26,222 registered students; while the selected private university has 5 colleges and approximately 3500 registered students. Ethical clearance was obtained from the Faculty of Medicine and Health Sciences Ethical Review Committee Board, Universiti Putra Malaysia (UPM/TNCPI/RMC/JKEUPM/1.4.18.1/F1).

### 2.2. Study Design

The study employed a cross-sectional study design.

### 2.3. Study Population

Simple random sampling was used for the selection of students. The list of students was obtained from the registration unit of the chosen faculties. Then, a computer random number generator was used to select the students recruited for the study. This simple random technique was employed to avoid any potential biases. The purpose of the study was given to the selected students before their class sessions, and they were encouraged to participate. Selected students who were present at the time of the survey were approached by the investigators. Those who agreed to participate were asked to give their consent and complete a set of questionnaires. The questionnaires were designed in English, but translated to the local dialect (Bahasa Malaysia) and were distributed using hard copies. The students were asked to put the questionnaire in a box placed at the entry of each faculty to ensure confidentiality and to increase the rate of participation. All undergraduate and postgraduate students from health (medicine, biomedicine, nursing and nutrition) and non-health (computer science and information technology) programs who were present during the survey were included in the study. Students who were absent due to semester breaks or on medical leaves during the period of data collection were exempted from the study.

### 2.4. Instrument

The study instrument was a self-administered questionnaire that was modified from the International AIDS Questionnaire-English Version (IAQ-E) [11] and from other relevant KAP studies on STDs [12]. The content validity of the study instrument was done by cross-checking and authentication from experts in the field of study. Afterwards, modifications were made according to the recommendations and counsel. Accordingly, the questionnaire was pre-tested on 20 students of 19–28 years old as part of a pilot study, and a few adjustments were made by changing the question wording.

The validated questionnaire was made up of four parts: socio-demographic characteristics (gender, age, ethnicity, educational level, religion and faculty type). The students’ knowledge on STDs was assessed by using a 14-item questionnaire (knowledge on the different types of STDs, their causes, transmission routes, preventive practices, symptoms and complications of STDs). Participants were instructed to give a “yes” or “no” answer. Each right and wrong response was given a score of 1 and 0, respectively. Those students with knowledge scores above the mean were categorized as having good knowledge, while those with knowledge scores below the mean were categorized as having poor knowledge.

Attitude related to STDs was assessed using a 20-item questionnaire (attitudes towards STD screening, self-perceived risk, perception on the necessity of condom use and treatment seeking attitudes). The responses were classified based on a 4-point Likert scale where 1, 2, 3 and 4 were used for “strongly disagree”, “disagree”, “agree” and “strongly agree”, respectively. All results were discussed by descriptive data analysis.

The preventive practice questions contained 6 items (sexual abstinence, condom use, having sex with only one partner, receiving screened blood and STD testing). Respondents were asked to give a response of “yes” or “no”. Each “yes” response was scored “1”, while a “no” response was scored “0”. The mean preventive score was obtained, and those students that had scores above the mean were categorized as having “acceptable preventive practice”, while those with scores lower than the mean were categorized as having “unacceptable preventive practice”. For the item “Do you abstain from sex”, it was interpreted as always abstaining from sex. In addition, students who answered “no” to this question were instructed to proceed further by answering the remaining items on preventive practices. Meanwhile, for the item “Do you have sex with only one partner for the past 12 months”, it was intended to examine whether the students had multiple sexual partners within that period. Specific risky behaviors were assessed among sexually-active students. Sexually active was defined as those who had sexual intercourse in the past three months or more [13]. Sexual abstinence was considered a student who has never had sexual intercourse with a partner in a lifetime.

### 2.5. Data Analysis

Data were analyzed using SPSS Version 21 statistical software (SPSS Inc., Chicago, IL, USA). Descriptive statistics were used to determine the frequency of the students’ socio-demographic characteristics, as well as their KAP scores. Chi square was used to analyze the association between categorical variables. In all cases, a *p*-value < 0.05 was considered to be statistically significant at 95% CI. Logistic regression was used for the respective variables with significant associations.

## 3. Results

### 3.1. Demographic Data

Seven hundred out of 748 students participated in this study, giving a response rate of 94%. The majority were females (63.6%), while the remaining were males (36.4%). The age group of the participants ranged from 17 to 30 years old with the mean age of 22.1 (±2.8). The mean age at sexual debut was 18.2 (±3.1). The participants within the age group of 17–23 years were the highest population involved (69.1%) as compared to the age group of 24–30 years old. Most of them were undergraduates (78.3%), while only 21.7% were postgraduates. Malays (53%) were more predominant compared to Chinese (19.6%), Indian (9.3%) and others (18.1%). With regard to religion, Muslim (58.6%) was the predominant religion embraced by them, followed by Catholic (17.3%), Buddhist (14.7%) and Hindus (9.4%). In addition, 57.1% and 42.9% of the participants were from non-health sciences and health sciences faculties, respectively. The socio-demographic characteristics of the participants are shown in the Supplementary Materials (Table S1).

### 3.2. KAP on STDs and Risky Behaviors

#### 3.2.1. Source of Information on STDs

With regard to the sources of information, most (77.3%) of the students identified the Internet as the main source. More than half of the students obtained it from the faculty curriculum. Only 34.0% of them obtained the information on STDs from their family members. Sources of information are shown in the Supplementary Materials (Table S2).

#### 3.2.2. Knowledge on STDs

The mean knowledge score was 23.3 (±8.4). The majority of them (86.6%) had heard about STDs. HIV/AIDS (83.6%) was best known among STDs, followed by syphilis (63.9%). Less than half (45.4%) of the students knew that gonorrhea was an STD. Chlamydia (26.0%), hepatitis B (25.1%), hepatitis C (22.0%) and trichomoniasis (21.0%) were poorly known as STDs by the students. Surprisingly, tuberculosis and asthma were known as STDs by 61.9% and 49.9% of the students, respectively. Virus (77.4%) was mostly known as the causative organism of STDs by the students followed by bacteria (66.3%). Only 22.6% of them thought that parasites were the causative organisms. Surprisingly, 37.4% of them thought mosquito was the causative organism of STDs. The result showed that almost all students (92.9%) knew that sexual intercourse is a route of STD transmission. A large proportion of them (76.4%) knew that use of condoms could reduce the risk of infection. Almost all of the students (88.1%) knew that having multiple sexual partners could increase the risk of infection.

Concerning symptoms of STDs, 65.4% of the students knew that pain while passing out urine is a symptom, while only 17.0% of them knew that sore throat is also a symptom of STD. Half (50.4%) of them knew that a person could have STDs without any visible symptoms. Cervical cancer was mostly known as a complication of STDs by 67.0% of the students, but only a few of them (34.1%) knew that ectopic pregnancy could be a complication. Table 1 shows the participants’ knowledge on STDs.

#### 3.2.3. Attitudes on STDs

Regarding the importance of condoms, 15.3% and 48.4% of the students strongly agreed and agreed, respectively, that condoms could protect people from STDs. In addition, 41.7% of them disagreed that condoms are not necessary for anal sex. More than half (54.2%) of them strongly disagreed that numerous sexual partners play no role in STDs transmission. Meanwhile, 63.8% of the students strongly felt that they are worried about contracting STDs. Interestingly, high percentages of students strongly agreed to seek treatment immediately if they (85.5%) and their partners (87.4%) have symptoms of STDs. Table 2 shows the participants’ attitudes related to STDs.

#### 3.2.4. Practices and Risky Behaviors towards STDs

With regard to sexual activity, 80.0% of students never had any sexual intercourse in their lifetime. However, 20.0% of students had experienced sexual intercourse and were sexually active. Table 3 shows the students’ preventive practices towards STDs. For these sexually-active students, less than half (41.0%) of them used a condom the last time they had sex. Surprisingly, the majority (66.7%) of the students had multiple sexual partners for the past 12 months (Table 4). Moreover, a majority of the students (65.9%) had watched pornographic materials, while 18.0% of them had sex with commercial sex workers. Meanwhile, 17.4% and 5.8% of them took alcohol and injected drugs before having sex, respectively (Table 4).

### 3.3. KAP Analysis with Other Parameters

Table 5 shows the associations between the students’ knowledge level and their socio-demographic characteristics. A higher level of good knowledge was observed among females than males (53.0% vs. 44.3%; prevalence ratio (PR) = 0.836, 95% CI = 0.710–0.984). Students in the age group of 24–30 years old had a higher level of good knowledge compared to the younger age group (57.9% vs. 46.4%; PR = 0.801, 95% CI = 0.691–0.930). As expected, postgraduates had a higher level of good knowledge compared to undergraduates (58.6% vs. 47.4%; PR = 0.810, 95% CI = 0.690–0.951). Students from health sciences possessed a higher level of good knowledge of STDs than non-health sciences (72.3% vs. 33.0%; PR = 2.192, 95% CI = 1.875–2.562). By logistic regression, students within the age group of 24–30 years old had 0.6-times higher odds of having good knowledge compared to the younger age group (AOR = 0.57, 95% CI = 0.377–0.859), and faculty type was a significant predictor where health sciences faculties had 5.7-times higher odds of having good knowledge compared to their counterparts (AOR = 5.69, 95% CI = 4.019–8.057). The logistic regression analysis is shown in the Supplementary Materials (Table S3). Religion and ethnicity were not statistically significant (*p*-values > 0.05). In addition, there were no significant associations observed between the preventive practices and socio-demographic profiles and knowledge level (Supplementary Materials, Table S4).

## 4. Discussion

Over the years, the devastating health, social and economic consequences of STDs have become a shocking reality globally especially among young people [14,15]. WHO has reported that the largest number of new curable cases of STDs was mainly from the South and Southeast Asia regions [16]. Thus, there is a need to embark on KAP studies on STDs among these young people so that the millennium development goal as proposed by WHO can be achieved by several strategies and interventions [17]. The purpose of using health and non-health sciences students in this study was to investigate whether these students would have good knowledge, attitudes and preventive practices towards STDS, which in turn could be the reliable “STD ambassadors” for the community in the future.

### 4.1. Knowledge of STDs on Etiological Agents, Modes of Transmission, Clinical Symptoms, Preventive Measures and Its Complications

A high proportion of university students in the present study have heard of at least one STD (86.6%). HIV/AIDS remains the most common type of STD, as documented by 83.6% of them. Similar findings were documented among youths (90%), as well as school pupils and university/college students (96%) by a few local studies [6,18]. This is not surprising, as awareness and intervention programs of HIV/AIDS have been continuously implemented by the Ministry of Health in Malaysia [19]. Similar findings have been reported in other countries. For instance, in Uganda and Nigeria, 89% and 91% of non-medical university students and undergraduates had heard of AIDS, respectively [20,21]. In Germany, their adolescents were reported to have low levels of knowledge and awareness of STDs with the exception of HIV/AIDS [22]. Knowledge on the other two common STDs, such as syphilis and gonorrhea, was known by 63.9% and 45.4% of our students in the present study. In contrast, higher percentages of knowledge on syphilis (95%) and gonorrhea (83%) were reported among Chinese university students in Guangdong, China [23]. This could be explained by the greater awareness among their students as Guangdong was reported to have the highest morbidity from syphilis and gonorrhea in China. We believe that this area might have continuous campaigns or health promotions on these diseases by the local authority. Surprisingly, almost less than a quarter of our students revealed that chlamydia, hepatitis B and hepatitis C are STDs in the present study. Chlamydia is commonly associated with gonorrhea, and in fact, a person with gonorrhea would require further tests and therapy for chlamydia, as reported by Lyss et al. [24]. Trichomoniasis, which is caused by a flagellated protozoon, has been listed as the least known by the students in the present study. This is further supported by only 22.6% of them knowing that parasites are one of the STD agents. To our knowledge, the least common STDs have been poorly recognized by Malaysian youths for more than a decade [6,8]. It has been observed that campaigns or health promotions for uncommon types of STDs have always been neglected by policy makers. Moreover, information about these uncommon STDs has been less emphasized in the current teaching modules which are mainly based on didactic lectures in many medical faculties in Malaysia. This notwithstanding, several studies have reported similar findings among their students [20,22,23,25].

Ironically, tuberculosis, which is caused by *Mycobacterium tuberculosis* and is considered endemic in Malaysia, was wrongly known as an STD by a large proportion of our students (61.9%) compared to a study in Turkey, which reported only 8% [25]. More surprisingly, asthma, a hypersensitivity condition affecting the lung, was also known as an STD, and mosquito (37.4%) was known as a causative organism of STDs by our students. These misconceptions were also observed among Malaysian school students in which leukemia and rheumatoid arthritis were perceived as STDs in a previous study [8]. Thus, a gap of knowledge on STDs still exists in our young population. Lack of knowledge and misconception about other STDs would place an individual at risk of sexual consequences [26].

As expected, sexual intercourse was identified as the main route of transmission by most students (92.9%) in the present study. Similar findings were observed in other studies [21,24,26]. However, slightly higher knowledge was observed among our students for kissing as a route of transmission (47.0%) compared to 28.1% of adolescences in Turkey [25]. Genital herpes can be transmitted by both herpes simplex virus 1 (HSV-1) and herpes simplex virus-2 (HSV-2) if a person is in contact with fluids in the lesions via fellatio or cunnilingus [27]. To our surprise, sharing clothes and sharing food/drinks were mistakenly perceived as the modes of transmission by students in the present study. These misconceptions would lead to stigma issues towards those who are living with HIV/AIDS, for instance.

As for the knowledge on the prevention of STDs, large proportions of students in the present study knew a condom (76.4%) could reduce the risk of acquiring STDs. This finding corroborates other studies. For instance, 93.5% and 78.7% Ugandan university students and Malaysian young males, respectively, knew that a condom is a preventive method. On the contrary, the misconception of monogamy as the preventive method was observed in 91.5% and 65.4% of their study populations [6,20]. Similarly, most (80%) of our students in the present study also thought monogamy is protective, and 44.7% of them thought the use of contraceptive pills could prevent STDs. Sexual abstinence was regarded as the most effective approach (71.4%) by our students, although studies have proven that there were no significant impacts on the STD rates among adolescents [28,29]. Knowledge on the risky behaviors revealed that the majority (88.1%) of our students knew having multiple sexual partners was unsafe, and almost half (51.6%) of our students understood using drugs would put them at risk in the present study. Similar percentages of knowledge on multiple sexual partners were reported in many studies [20,23], but a few reported slightly lower ones [8]. Contradictorily, a high rate (98.3%) of knowledge on drugs as a risk factor among young Turkish people in Capriot was observed [25]. A worrying fact is to note that only 38.6% of students knew alcohol intake was associated with STDs in the present study compared to 73% of non-medical university students in Makerere University, Uganda [20]. Consuming alcohol and drugs would impair one’s judgement on the risk of unprotected sexual behaviors [30,31].

Only half (50.4%) of the students in the present study knew that people with STDs would have no symptoms. This is worrying as a lack of this information would allow any potential outbreaks of STDs and its possible consequences among students [32]. A comparatively higher level of awareness (67%) on this matter was reported among high school students in Germany [22]. In the present study, most (82.9%) of the students were unaware of the fact that a sore throat is a symptom of STDs. Gonococcal pharyngitis presents with sore throat following oral sex with an infected partner [33]. With regard to complications, cervical cancer (67%) was highly known by the students, followed by infertility (60%) in the present study. This could be related to the increased awareness on human papillomavirus (HPV) vaccination among the students as a preventive measure of cervical cancer since the introduction of the HPV vaccination program in schoolchildren by the Ministry of Health in the year 2010 [34]. Lower percentages of awareness on cervical cancer caused by HPV were demonstrated by many studies [22,35,36]. Ectopic pregnancy (34.1%) was less regarded as a complication by our students in the present study. This could be related to the poor knowledge on gonorrhea and chlamydia as STDs among our students. Since questions were not specifically asked for each type of STD in the present study, students might have difficulties in remembering all information about STDs (recall bias). For instance, students were asked to respond to the question on the routes of transmission of STDs based on a few different options in our questionnaire. Our intention was to investigate the general knowledge of various routes of transmission of STDs. Undeniably, sexual route is the main route of transmission of acquiring STDs. However, since STDs are caused by many different types of microorganisms and the sexual route is not the only means of transmission, students might not be aware of the detailed information about each type of STD. Thus, they could not give their responses correctly. Moreover, social desirability bias could have influenced the results as issues related to sex and STDs are still considered social taboos among Malaysians, and the subjects could not be discussed openly. Thus, this could limit the participation rate. However, since the study was aimed at future healthcare providers, results from the present study could be used to design proper educational programs and interventions on STDs.

### 4.2. Attitudes, Risky Behaviors and Preventive Practices on STDs

Despite the students possessing moderate levels of knowledge in the present study, it is shocking to know that their preventive practices were poorly implemented. Among the sexually-active group, only 41% and 66.7% of them had used a condom and encountered multiple sexual partners, respectively. Similar findings were observed locally and abroad [8,22]. Multiple sexual contacts are a well-known behavioral risk factor for acquiring STDs [37]. It is quite astonishing to find that sexually-active students had injected drugs (5.8%), taken drugs (9.4%) and consumed alcohol (17.4%) during their sexual intercourse in the present study. Most (79.5%) of the students in the present study agreed that watching/reading pornographic materials could contribute to risky sexual practices. However, the majority of sexually-active students (65.9%) in the present study had watched those materials. Similar findings were documented on the pornographic exposure in China (74%) and Hong Kong (50.4%) [38,39]. Similarly for STDs screening, 88.8% of our students felt it was good, but only 21.7% of them had STDs tested annually. It is well known that having sufficient knowledge alone does not always guarantee a person to have appropriate behavior [40]. Although only 20% of students in the present study were sexually active, information on their risky behaviors is of great concern. The figure in the present study may be an underestimation of their sexual behavior, since the data were self-reported by the students. Moreover, the closed question format that was used in the present study could promote the risk of guesswork. It is well known that the risky behaviors of university students are influenced by various factors, such as their peers. Peers have an impact on risky behaviors, such as drinking alcohol, using drugs and watching pornographic movies [41]. It is of concern that these students might be able to influence other students to do these unacceptable behaviors. Thus, there is a need to initiate the risky sexual behavior prevention program among students in the future. Meanwhile, most students in the present study wanted the academic institutions to discuss issues on STD prevention. Similar finding was reported in Italy when almost all (95%) students highlighted that school should play an important role in sex education [42]. Similar scenarios have been reported elsewhere [23,43]. Since Malaysian youths are more likely to be involved in premarital sex due to the influence of mass media, rapid development of the economy and degradation of traditional values, as reported by Zulkifli et al. [44], there is an urgent need for higher authorities in the institution to advocate new or improve the existing curriculum or policy on STDs.

### 4.3. Associations between Knowledge and Preventive Practices of STDs with Sociodemographic Factors

In general, it is interesting to note that females in the present study were significantly knowledgeable and had positive attitudes. Our findings are consistent with a few studies [22,45,46], but another study reported the opposite [18]. However, males are equally at risk of acquiring and transmitting STDs; thus, this finding needs to be addressed accordingly. The knowledge of STDs was also significantly increased with age and among postgraduates. Students in the age group of 24–30 years old were 0.6-times more likely to have good knowledge than 17–23 years old in the present study. This might be explained by the fact that older students are more experienced in life, and they have been exposed to basic information regarding STDs. On the other hand, being comparatively young and lacking knowledge, these students would have more risky behaviors and exposure to pornographic materials despite having good sexual education at schools [38]. Sex education in Malaysian schools is still new and has not been standardized in terms of the mode of teaching delivery and the involvement of teachers or experts in the subject. We believe that it is high time for the Ministry of Education to review or evaluate the effectiveness of sex education in our school curriculum so that a better approach can be implemented. Meanwhile, our findings are inconsistent with a study that reported that religion was significantly associated with knowledge [8]. Nonetheless, it can be proposed that moral or religious values could be incorporated for the holistic approach of STD education or program in universities. Faculty type is also the predictor for knowledge level, and health sciences students were 5.7-times more likely to have good knowledge than non-health sciences students. This is not surprising as most of the medical faculties have already included the STD subjects in their curricula. Nonetheless, in view of the lack of knowledge on STDs in some aspects among the students in this study, the current modes of teaching need to be revised. Most of the medical faculties nowadays have changed their teaching methodologies into student-centered approaches coupled with a few innovative teaching techniques, such as blended learning. It is hoped that with such approaches, students would be able to acquire their knowledge at their own pace by the integration of online and face-to-face environments and can discuss their sexual issues openly.

### 4.4. Limitations

Our study has several limitations. Since this is a cross-sectional study, several independent variables could not be evaluated for the cause and effect associations. The results obtained in this study should not be generalized to all university students in Malaysia, as only three faculties were involved in the central zone. Moreover, a proportion of non-health sciences students are staying outside the university compared to health sciences students who are staying in hostels, which might have greater chances for risky sexual behaviors. The recent study should have involved school or university dropouts. Since many of them are less educated and jobless, they would be prone to be involved in more risky sexual behaviors. Thus, significant data can be obtained. The attitude components were not evaluated for each domain; thus, the findings are merely descriptive. Finally, since discussion about sex in Malaysia is still considered as a taboo, some questions related to sexual behaviors might be under-reported.

## 5. Conclusions

This study has described the knowledge, attitudes, risky behaviors and preventive practices of Malaysian university students towards STDs. Based on our findings, there is a critical need to re-evaluate the current sex education program and methods of teaching delivery in all schools and universities, which are mainly based on a recall basis. There is still a gap of knowledge pertaining to the non-HIV causes of STDs and the complications and clinical symptoms among our students, which should carefully be addressed by the universities’ stakeholders as a basis for the improvement of health promotion. In addition, health screening on STDs and their treatment interventions should be made available. However, future studies are needed to evaluate the cost-effectiveness of these implementations by each institution. Such implementations also need to be extended to young adults who do not attend colleges or universities, as well. Modifying the current teaching on STDs in medical curricula is urgently needed to ensure the STD ambassador is well equipped with sound knowledge, good attitudes and preventive practices, can actively participate in solving any sexual issues during his/her learning activities and, finally, can influence or empower other people to do the same. Without such efforts, it seems very difficult for us to produce a good healthcare provider with respect to STDs in the future.

## Figures and Tables

**Table 1:** University students’ knowledge of sexually-transmitted diseases.

Question	Correct *n* (%)	Incorrect *n* (%)
Have you ever heard of STDs?	606 (86.6)	93 (13.4)
Which of the following is an STD?	-	-
Gonorrhea	318 (45.4)	382 (54.6)
Syphilis	447 (63.9)	253 (36.1)
Genital herpes	352 (50.3)	348 (49.7)
Trichomoniasis	147 (21.0)	553 (79.0)
Tuberculosis	267 (38.1)	433 (61.9)
Asthma	351 (50.1)	349 (49.9)
HIV/AIDS	585 (83.6)	115 (16.4)
Chlamydia	182 (26.0)	518 (74.0)
Hepatitis B	176 (25.1)	524 (74.9)
Hepatitis C	154 (22.0)	546 (78.0)
What are the causative organisms of STDs?	-	-
Bacteria	464 (66.3)	236 (33.7)
Virus	542 (77.4)	158 (22.6)
Fungi	271 (38.7)	429 (61.3)
Parasites	158 (22.6)	541 (77.3)
Mosquitoes	438 (62.6)	262 (37.4)
What are the routes of transmission of STDs?	-	-
Sexual intercourse	650 (92.9)	50 (7.1)
Blood transfusion	456 (65.1)	244 (34.9)
Sharing injection needles	477 (68.1)	223 (31.9)
Sharing food/drinks	404 (57.7)	296 (42.3)
Sharing clothes	258 (36.9)	442 (63.1)
Infected mother to child	420 (60.0)	280 (40.0)
Kissing	329 (47.0)	371 (53.0)
Use of contraceptive pills can reduce risk of STDs	387 (55.3)	313 (44.7)
Use of condoms can decrease the risk of being infected with an STD *	534 (76.4)	165 (23.6)
Monogamy can reduce one’s chance of infection	560 (80.0)	140 (20.0)
Alcohol intake can increase an individual’s susceptibility to STDs	270 (38.6)	430 (61.4)
Intake of some drugs can increase an individual’s susceptibility to STDs	361 (51.6)	339 (48.4)
Having multiple sexual partners can increase chances of being infected	617 (88.1)	83 (11.9)
Sexual abstinence is the most effective means of avoiding STDs	500 (71.4)	200 (28.6)
What are the symptoms of STDs?	-	-
Ulcers in the genital organ	438 (62.6)	262 (37.4)
Pain while passing out urine	458 (65.4)	242 (34.6)
Swollen glands, fever and body ache	375 (53.6)	325 (46.4)
Discharge from the penis	446 (63.7)	254 (36.3)
Discharge from the vagina	437 (62.4)	263 (37.6)
Itching around the vagina	441 (63.0)	259 (37.0)
Sore throat	120 (17.1)	580 (82.9)
Painless sores on the mouth and genital area	296 (42.3)	404 (57.7)
Can people with STDs have no symptoms?	353 (50.4)	347 (49.6)
What are the complications of STDs?	-	-
Infertility	420 (60.0)	280 (40.0)
Cervical cancer	469 (67.0)	231 (33.0)
Body weakness	366 (52.3)	334 (47.7)
Ectopic pregnancy	239 (34.1)	461 (65.9)
Still birth	322 (46.0)	378 (54.0)

* Only one student did not answer this question completely.

**Table 2:** Attitudes on sexually-transmitted diseases among university students.

Item	Strongly Disagree *n* (%)	Disagree *n* (%)	Agree *n* (%)	Strongly Agree *n* (%)
I feel condoms protect people against STDs	70 (10.0)	183 (26.2)	338 (48.4)	107 (15.3)
I feel it is not necessary to use condom during anal sex	270 (38.7)	291 (41.7)	105 (15.0)	32 (4.6)
If both partners are infected with STDs, I feel there is no need of using a condom	242 (34.6)	243 (34.8)	153 (21.9)	61 (8.7)
I feel numerous sexual partners play no role in STDs transmission	378 (54.2)	174 (24.9)	99 (14.2)	47 (6.7)
I feel condoms play an important role in preventing STDs	70 (10.0)	176 (25.2.)	320 (45.8)	132 (18.9)
I feel it is not necessary for academic institutions to discuss issues regarding prevention of STDs	372 (53.3)	208 (29.8)	64 (9.2)	54 (7.7)
I feel banning of prostitution can control the spread of STDs	56 (8.0)	90 (12.9)	205 (29.4)	34.7 (49.7)
I feel screening for STDs is good	25 (3.6)	53 (7.6)	255 (36.5)	365 (52.3)
I feel screening for STDs before marriage is important	31 (4.4)	41 (5.9)	201 (28.8)	425 (60.9)
I think watching/reading pornographic materials can contribute to risky sexual practices	51 (7.3)	92 (13.2)	237 (34.1)	316 (45.4)
STDs are not dangerous because they can be cured	310 (44.4)	264 (37.8)	97 (13.9)	27 (3.9)
In my opinion, I feel STDs can cause death if left untreated	44 (6.3)	57 (8.2)	296 (42.3)	302 (43.2)
I am worried about contracting STDs	0 (0)	107 (15.4)	145 (20.8)	445 (63.8)
The STD problem is something that I have not given much thought to	301 (43.1)	214 (30.7)	120 (17.2)	61 (8.7)
Homosexual men are solely to be blamed for the spread of STDs	158 (22.6)	273 (39.1)	184 (26.4)	83 (11.9)
If I have unprotected sexual intercourse, I am most concerned about:	-	-	-	-
*Getting HIV*	28 (4.0)	34 (4.9)	219 (31.5)	415 (59.6)
*Getting STDs aside from HIV*	29 (4.2)	82 (11.8)	240 (34.5)	342 (49.2)
*Unwanted pregnancy*	49 (7.0)	87 (12.5)	198 (28.4)	360 (51.7)
If I notice symptoms of STDs, I think I should seek treatment immediately	8 (1.1)	18 (2.6)	75 (10.8)	596 (85.5)
If I notice symptoms of STDs in my partner, I will advise him/her to seek treatment immediately	2 (0.3)	8 (1.1)	78 (11.2)	609 (87.4)

**Table 3:** Preventive practices on sexually-transmitted diseases among university students.

Preventive Practices ^†^	Yes *n* (%)	No *n* (%)
Do you abstain from sex?	560 (80.0)	140 (20.0)
Was a condom used the last time you had sex?	57 (41.0)	82 (59.0)
Do you have sex with only one partner for the past 12 months?	46 (33.3)	92 (66.7)
If you ever received a blood transfusion, was the blood screened?	6 (42.9)	8 (57.1)
Do you get tested for STDs annually?	30 (21.7)	108 (78.3)
Does your partner get tested for STDs annually?	19 (13.8)	119 (86.2)

^†^ The mean (SD) preventive practice score was 2.1 (±1.0).

**Table 4:** Risky behaviors among the sexually-active university students.

Item *	*N*	Yes (%)	No (%)
Do you inject drugs before having sex?	139	8 (5.8)	131 (94.2)
Do you take drugs before having sex?	138	13 (9.4)	125 ( 90.6)
Do you drink alcohol before having sex?	138	24 (17.4)	114 ( 82.6)
Do you share injection needle with others?	138	4 (2.9)	134 ( 97.1)
Do you read pornographic materials?	138	91 (65.9)	47 (34.1)
Do you have sex with commercial sex workers?	139	25 (18.0)	114 (82.0)

* 1–2 students did not complete the questionnaire.

**Table 5:** Associations between university students’ knowledge level and their socio-demographic characteristics.

Variables	Knowledge Level		*n* (%)	x^2^	*p*	Prevalence Ratio (CI) ^#^
	Good (%)	Poor (%)				
Gender (*n* = 700)	-	-	-	-	-	-
Male	113 (44.3)	142 (55.7)	255 (36.4)	4.931	0.026 *	0.836 (0.710–0.984)
Female	236 (53.0)	209 (47.0)	445 (63.6)	-	-	
Age Group (*n* = 699) ^†^	-	-	-	-	-	-
17–23	224 (46.4)	259 (53.6)	483 (69.1)	7.887	0.005 *	0.801 (0.691–0.930)
24–30	125 (57.9)	91 (42.1)	216 (30.9)	-	-	
Educational Level (*n* = 699)	-	-	-	-	-	-
Undergraduate	260 (47.4)	288 (52.6)	548 (78.3)	5.872	0.015 *	0.810 (0.690–0.951)
Postgraduate	89 (58.6)	63 (41.4)	152 (21.7)	-	-	
Faculty Type (*n* = 700) ^†^	-	-	-	-	-	-
Health Sciences	217 (72.3)	83 (27.7)	300 (42.9)	10.608	0.001 *	2.192 (1.875–2.562)
Non-Health Sciences	132 (33.0)	268 (67.0)	400 (57.1)	-	-	
Ethnicity (*n* = 700)	-	-	-	-	-	-
Malay	188 (50.7)	183 (49.3)	371 (53.0)	0.211	0.646	1.036 (0.892–1.202)
Non-Malay	161 (48.9)	168 (51.1)	329 (47.0)	-	-	
Religion (*n* = 700)	-	-	-	-	-	-
Muslim	199 (48.5)	211 (51.5)	410 (58.6)	0.690	0.406	0.938 (0.808–1.089)
Non-Muslim	150 (51.7)	140 (48.3)	290 (41.4)	-	-	

* Significant result by chi-square test (*p* < 0.05); ^†^ significant results by logistic regression for the age group (*p* = 0.007) and non-health sciences (*p* = 0.001); ^#^ CI = confidence interval.

## Supplementary Materials

The following are available online at http://www.mdpi.com/1660-4601/14/2/159/s1, Table S1: Socio-demographic characteristics of the health and non-health university students in this study; Table S2: Sources from which the university students obtained their STD information; Table S3: Multiple logistic regression predicting university students’ knowledge level on STDs; Table S4: Association between preventive practices level and socio-demographic characteristics and knowledge level of the students related to STDs.
