# Supplementary material for: Assessing the Knowledge Level, Attitudes, Risky Behaviors and Preventive Practices on Sexually Transmitted Diseases among University Students as Future Healthcare Providers in the Central Zone of Malaysia: A Cross-Sectional Study
Source: Int J Environ Res Public Health. 2017 Feb 8;14(2):159. doi: 10.3390/ijerph14020159 (PMC5334713; doi:10.3390/ijerph14020159)
Supplement: Supplementary file 1 [file ijerph-14-00159-s001.pdf]

Supplementary Materials: Assessing the Level of Knowledge, Attitudes, Risky Behaviors and Preventive Practices on Sexually Transmitted Diseases among University Students as Future Health Care Providers in the Central Zone of Malaysia: A Cross-Sectional Study

Adigun Temiloluwa Folasayo, Afolayan John Oluwasegun, Suhailah Samsudin, Siti Nor Sakinah Saudi, Malina Osman and Rukman Awang Hamat

Table S1. Socio-demographic characteristics of the health and non-health university students in this study.

Variables	*N*	%
Gender	-	-
Male	255	36.4
Female	445	63.6
Age Group (in years) *	-	-
17–23	483	69.1
24–30	216	30.9
Ethnicity	-	-
Malay	371	53.0
Chinese	137	19.6
Indian	65	9.3
Others	127	18.1
Educational Level	-	-
Undergraduate	548	78.3
Postgraduate	152	21.7
Religion	-	-
Islam	410	58.6
Hindu	66	9.4
Buddhist	103	14.7
Christian	121	17.3
Faculty Type	-	-
Health Sciences	300	42.9
Non-Health Sciences	400	57.1

* Only one student did not complete this information.

Table S2. Sources from which the university students obtained their STD information.

Source	Frequency	%
Internet	541	77.3
Newspaper/Magazine	446	63.8
Television	396	56.6
Radio	227	32.4
Seminar	295	42.1
School curriculum	360	51.4
Family	238	34.0
Friends	325	46.4
Pamphlet	314	44.9
Signboard	240	34.3
Health Center	399	57.0

Table S3. Multiple logistic regression predicting university students' knowledge level on STDs.

Variable	β	*p*-Value	Adjusted Odds Ratio	95% CI
Constant	-	0.001	2.174	-
Gender	-	-	-	-
Male	0.027	0.882	1.027	0.722–1.460
Female	-			
Age group (in years)	-	-	-	-
17–23	−0.564	0.007 *	0.569	0.377–0.859
24–30	-			
Education level	-	-	-	-
Undergraduate	−0.205	0.380	0.815	0.515–1.788
Postgraduate	-			
Faculty type	-	-	-	-
Health Sciences	1.739	0.001 *	5.690	4.019–8.057
Non-Health Sciences	-			

Note: Method = enter; R^2 = 21.5%; overall percentage = 69.2%; * significant at $p < 0.05$.

Table S4. Association between preventive practices level and socio-demographic characteristics and knowledge level of the students related to STDs.

Variables	Practices Level (%)		*n* (%)	x^2	*p*	Prevalence Ratio (C.I)
-	Acceptable	Unacceptable	-	-	-	-
Gender (*n* = 138)	-	-	-	-	-	-
Male	23 (26.4)	64 (73.6)	87 (62.6)	0.231	0.631	0.876 (0.511–1.501)
Female	16 (30.2)	37 (69.8)	53 (37.4)	-	-	-
Age group (*n* = 139)	-	-	-	-	-	-
17–23	18 (31.6)	39 (68.4)	57 (41.0)	0.663	0.416	1.363 (0.733–1.137)
24–30	21 (25.3)	62 (74.7)	83 (59.0)	-	-	-
Educational Level (*n* = 139)	-	-	-	-	-	-
Undergraduate	25 (31.3)	55 (68.8)	80 (56.8)	1.069	0.301	1.339 (0.763–2.350)
Postgraduate	14 (23.3)	46 (76.7)	60 (43.2)	-	-	
Faculty Type (*n* = 139)	-	-	-	-	-	-
Health Sciences	8 (25.8)	23 (74.2)	31 (21.6)	0.083	0.773	0.907 (0.466–1.768)
Non-Health Sciences	31 (28.4)	78 (71.6)	109 (78.4)	-	-	-
Ethnicity (*n* = 138)	-	-	-	-	-	-
Malay	14 (35.0)	26 (65.0)	40 (28.8)	1.422	0.233	1.400 (0.814–2.407)
Non-Malay	25 (25.0)	75 (75.0)	100 (71.2)	-	-	-
Religion (*n* = 139)	-	-	-	-	-	-
Muslim	22 (31.4)	48 (68.6)	70 (50.0)	0.889	0.346	1.294 (0.755–2.219)
Non-Muslim	14 (24.3)	53 (75.7)	70 (50.0)	-	-	-
Knowledge Level (*n* = 139)	-	-	-	-	-	-
Good	18 (25.0)	54 (75.0)	71 (51.1)	0.602	0.438	1.235 (0.723–2.110)
Poor	21 (30.9)	47 (69.1)	68 (48.9)	-	-	-

$p < 0.05$ was considered significant.

© 2017 by the authors. Submitted for possible open access publication under the terms and conditions of the Creative Commons Attribution (CC-BY) license (http://creativecommons.org/licenses/by/4.0/).
